# Acetyl-CoA carboxylase inhibitors attenuate WNT and Hedgehog signaling and suppress pancreatic tumor growth

**DOI:** 10.18632/oncotarget.12650

**Published:** 2016-10-13

**Authors:** Elissaveta Petrova, Arne Scholz, Juliane Paul, Andrea Sturz, Katja Haike, Franziska Siegel, Dominik Mumberg, Ningshu Liu

**Affiliations:** ^1^ Bayer AG, Drug Discovery, Berlin, Germany; ^2^ Current address: Merck KGaA, Darmstadt, Germany

**Keywords:** ACC, WNT, Hedgehog, lipidation, PDAC

## Abstract

Acetyl-CoA carboxylase (ACC) is the rate-limiting enzyme in *de novo* fatty acid synthesis, and its ACC1 isoform is overexpressed in pancreatic and various other cancers. The activity of many oncogenic signaling molecules, including WNT and Hedgehog (HH), is post-translationally modified by lipidation. Here, we report that inhibition of ACC by a small molecule inhibitor, BAY ACC002, blocked WNT3A lipidation, secretion, and signaling. In pancreatic cancer cells, where WNT and HH are key oncogenic drivers, ACC inhibition simultaneously suppressed WNT and HH signaling, and led to anti-proliferative effects. Treatment with ACC inhibitors blocked tumor growth and converted the poorly differentiated histological phenotype to epithelial phenotype in multiple cell line-based and patient-derived pancreatic cancer xenograft models. Together, our data highlight the potential utility of ACC inhibitors for pancreatic cancer treatment, and provide novel insight into the link between upregulated *de novo* fatty acid synthesis in cancer cells, protein lipidation, and oncogenic signaling.

## INTRODUCTION

The majority of normal cells preferentially use circulating lipids, exogenously derived from the diet. Cancer cells, however, exhibit an increase in *de novo* fatty acid synthesis irrespective of exogenous lipid levels [1, 2]. While endogenously synthesized fatty acids are converted into phospholipids and utilized as building blocks for membranes during the rapid division of these cells [3, 4], lipids have also long been recognized as signaling molecules that trigger profound physiological responses directly, or through covalent or non-covalent binding to signaling proteins [5].

Among the covalent lipid modifications of proteins, palmitoylation has been shown to be important in regulating the secretion and activity of several oncogenic signaling molecules, such as WNT and HH [6–8]. Often these pathways are dysregulated together, and a prominent example of this is pancreatic ductal adenocarcinoma (PDAC), where WNT and HH proteins are frequently overexpressed during disease onset and progression [9]. In this paper, we used pancreatic cancer as a model system to show a novel therapeutic strategy to simultaneously block the activation of several oncogenic signaling pathways, such as WNT and HH, by suppressing palmitoylation of their ligands. This was achieved by inhibition of acetyl-CoA carboxylase (ACC), a rate-limiting enzyme in the *de novo* synthesis of lipids, whose isoform ACC1 is overexpressed in cancers [10, 11]. Inhibition of ACC by a potent and selective small molecule inhibitor BAY ACC002 (Liu *et al*., in preparation), resulted in a decrease in both HH and WNT signaling, as well as in cell proliferation *in vitro* and tumor growth *in vivo*, in a set of PDAC xenograft mouse models.

## RESULTS

### Inhibition of ACC blocks the lipidation, secretion and signaling of WNT3A

**Figure 1:**
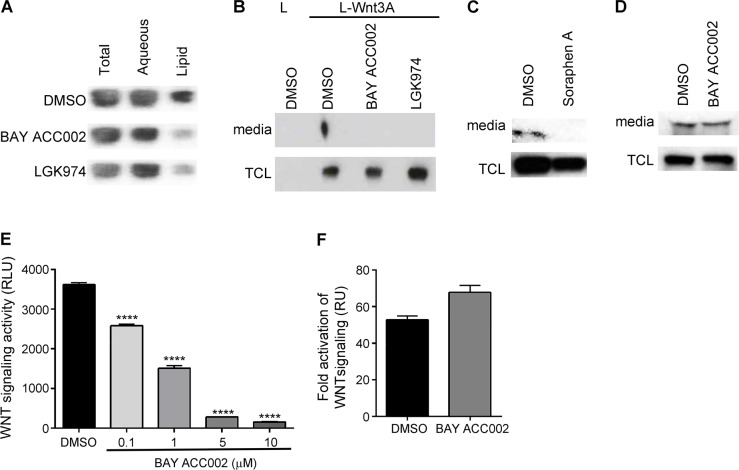
Inhibition of ACC Decreases Lipidation, Secretion and Signaling of WNT3A **A**. Amounts of lipidated and non-lipidated WNT3A in L-Wnt3A cells, transfected with a PORCN plasmid, and treated with DMSO, 10 μM BAY ACC002 or 5 μM LGK974 for 24 h. The cells were lysed and subjected to Triton X-114 phase separation. The amount of WNT3A which partitions in each layer was quantified by Western blot. **B**. Amount of WNT3A secreted from cells treated with DMSO, 5 μM BAY ACC002 or 1 μM LGK974 for 48 h. Amount of WNT3A in the media and in the total cell lysate (TCL) was quantified by Western blot. L cells were used as a control. **C**. Amount of WNT3A secreted from cells treated with DMSO or 5 μM Soraphen A for 48h. Amount of WNT3A in the media and in the TCL was quantified by Western blot. **D**. Amount of CYR61 secreted from Panc-1 cells, treated with DMSO or 5 μM BAY ACC002 for 48h. Amount of CYR61 in the media and the TCL was detected by Western blot. **E**. Signaling activity of WNT3A, secreted from L-Wnt3A cells, treated with DMSO or varying concentrations of BAY ACC002. Conditioned media from the cells was collected and HEK293-TOP cells were grown in the presence of the media for 24 h before luciferase levels were measured. **F**. Effect of BAY ACC002 on exogenous WNT pathway activation. HEK293-TOP cells were stimulated with 200 ng recombinant human WNT3A, and grown in the presence of DMSO or 10 μM BAY ACC002 for 48 h, followed by a measurement of luciferase levels. Each bar in (E) and (F) represents mean±SEM (*n* = 3, ****, *p* < 0.0001, Student's *t* test).

To investigate the direct effect that ACC inhibition has on protein lipidation, we used two recently identified potent and selective small molecule inhibitors of ACC - BAY ACC001 and BAY ACC002 - with predominant activity against ACC1 (IC_50_ = 0.28 μM and 0.10 μM, respectively) compared to ACC2 (IC_50_ = 2.6 μM and 1.4 μM), and without off-target activities in two Lead Profiler screens at 10 μM (Supplementary Figure S1A; Liu *et al*., in preparation). First, we explored the effect of ACC inhibition on WNT lipidation by transfecting L-Wnt3A cells (a transgenic mouse fibroblast cell line stably expressing WNT3A) with cDNA for the WNT acyltransferase Porcupine (PORCN), and then treating with BAY ACC002. Treatment with the ACC inhibitor led to reduction in the lipidated vs unlipidated WNT3A ratio from 82% to 36% as measured in a Triton X-114 phase separation assay, and the effect was comparable to the effect observed with the PORCN inhibitor LGK974, which blocks the transfer of palmitate onto WNT proteins (Figure 1A, Supplementary Figure S1B) [12].

Since lipid modification is required for both WNT secretion [6] and signaling [13], we measured the amount of WNT3A secreted in the media of L-Wnt3a cells, in order to functionally confirm the suppression of WNT3A lipidation by ACC inhibition. Treatment with both BAY ACC002 and Soraphen A (an allosteric ACC inhibitor) [14] blocked WNT3A secretion (Figure 1B, 1C, Supplementary Figure S1C). A similar result was observed with the PORCN inhibitor LGK974 (Figure 1B). Notably, inhibition of WNT secretion by BAY ACC002 appeared to be specific, as treatment with the compound had effect neither on L-Wnt3A cell growth (Supplementary Figure S1D) nor on protein secretion in general (e.g. secretion of the cysteine-rich angiogenic inducer 61(CYR61), Figure 1D).

To further confirm that there was indeed no functional palmitoylated WNT3A secreted from the L-Wnt3A cells treated with BAY ACC002, the conditioned media (CM) was harvested and used to stimulate HEK293 cells carrying a stably-transfected TOPFlash reporter plasmid (HEK293-TOP). CM from cells treated with BAY ACC002 revealed a dose-dependent reduction of reporter activity compared to control CM (Figure 1E). Importantly, BAY ACC002 had no effect on exogenous WNT3A-stimulated reporter activity (Figure 1F). Taken together, these data demonstrate that ACC inhibition could effectively suppress WNT signaling by blocking WNT protein lipidation and secretion.

### ACC inhibition suppresses WNT and HH signaling in pancreatic cancer cells in vitro

**Figure 2:**
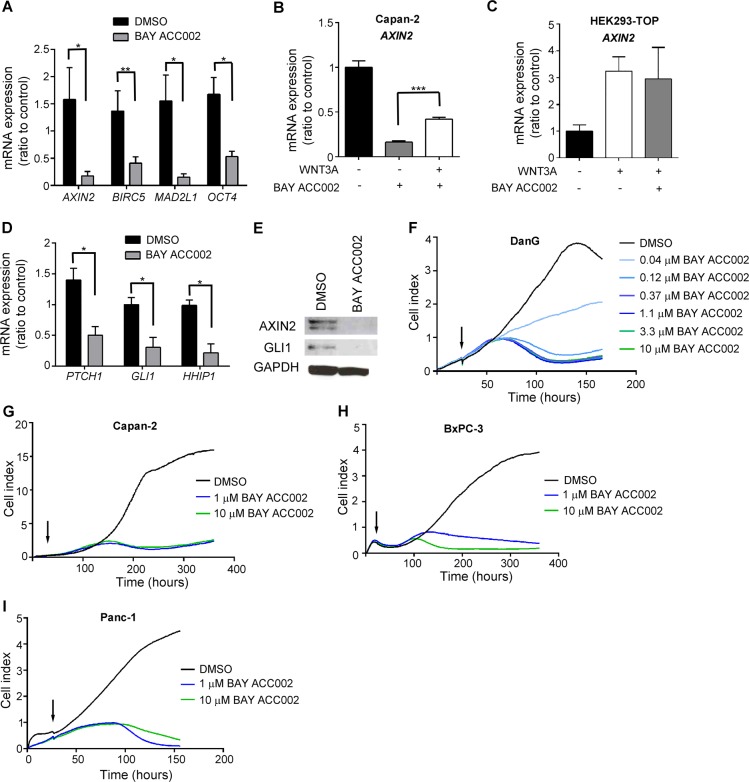
ACC Inhibition Decreases WNT and HH Signaling, and Reduces Proliferation in Pancreatic Cancer Cells *In Vitro* **A**. Expression of β-catenin target genes in Capan-2 cells after treatment with DMSO or 10 μM BAY ACC002 for 72 h. RNA was extracted and the mRNA expression level of β-catenin target genes was determined by qRT-PCR. **B**. Capan-2 cells were treated 200ng recombinant human WNT3A, and grown in the presence of DMSO or 10 μM BAY ACC002 for 72 h. RNA was extracted and the mRNA expression level of *AXIN2* was determined by qRT-PCR. **C**. HEK293-TOP cells were stimulated with 200ng recombinant human WNT3A, and grown in the presence of DMSO or 10 μM BAY ACC002 for 72 h. RNA was extracted and the mRNA expression level of *AXIN2* was determined by qRT-PCR. **D**. Expression of HH target genes in Capan-2 cells, treated with BAY ACC002. Cells were treated as in (A) and mRNA expression levels were determined by qRT-PCR. **E**. Expression of AXIN2 and GLI1 in Capan-2 cells treated with DMSO or 10 μM BAY ACC002 for 96 h. The cells were then lysed and protein levels were detected by Western blot. GAPDH levels were monitored as a control. **F**. Growth curves of DanG cells, treated with varying concentrations of BAY ACC002 (arrow indicates time of drug addition). Cell growth was measured over time using the xCELLigence system. Experiments were performed in triplicate. **G**., **H**. and **I**. Capan-2, BxPC-3 and Panc-1 cells were treated with BAY ACC002, and cell growth over time was determined as in (G). Each bar in (A) - (D) represents mean±SEM (*n* = 3-4 for (A), *n* = 2-3 for (B)-(D), *, *p* < 0.05, **, *p* < 0.01, ***, *p* < 0.001, Student's *t* test).

To investigate the effects of ACC inhibition on WNT and HH signaling, we chose pancreatic cancer as a model system, since PDAC is often characterized by upregulated ACC1 expression (Supplementary Figure S2A) and disregulated WNT and HH pathways. Treatment of Capan-2, a pancreatic cancer cell line with autocrine WNT and HH signaling, with BAY ACC002, suppressed the expression of the WNT/β-catenin target genes *AXIN2*, *BIRC5*, *MAD2L1* and *OCT4* (Figure 2A). Notably, this effect was partially rescued by addition of exogenous WNT3A (Figure 2B). At the same time, BAY ACC002 had no effect on exogenous WNT3A- stimulated *AXIN2* expression in HEK293-TOP cells (Figure 2C). Importantly, in Capan-2 cells, BAY ACC002 also significantly blocked the expression of the HH/GLI1 target genes *PTCH1, GLI1 and HHIP1* (Figure 2D). Likewise, it decreased expression of another HH/GLI1 target gene, *Secreted frizzled-related protein 1* (*SFRP1*), which was upregulated in pancreatic cancer (Supplementary Figures S2A, S2B). Meanwhile, treatment of these cells with the PORCN inhibitor LGK974 or the HH pathway inhibitor GDC-0449 [15], primarily decreased the expression of WNT or HH target genes, respectively (Supplementary Figure S2D). The effects of BAY ACC002 treatment were also confirmed at protein level, where decrease in the expression of both AXIN2 and GLI1 was observed (Figure 2E). Altogether, these data suggest that ACC inhibition could simultaneously suppress both WNT and HH signaling in pancreatic cancer cells *in vitro*.

### ACC inhibition blocks proliferation of pancreatic cancer cells

Next, we investigated the functional consequence of ACC inhibition by conducting a real-time cell growth analysis using the xCELLigence system in DanG, Capan-2, BxPC-3, and Panc-1 pancreatic cancer cells. Treatment with BAY ACC002 suppressed cell proliferation (Figure 2F-2I) - an effect also observed with the WNT acyltransferase inhibitor LGK974 in Capan-2 cells (Supplementary Figure S2E) or with an HH acyltransferase inhibitor in Panc-1 cells [16]. These data indicate that inhibiting ACC could effectively block cell proliferation *in vitro* in pancreatic cancer cells.

### ACC inhibition blocks WNT and HH signaling in pancreatic cancer models in vivo

**Figure 3:**
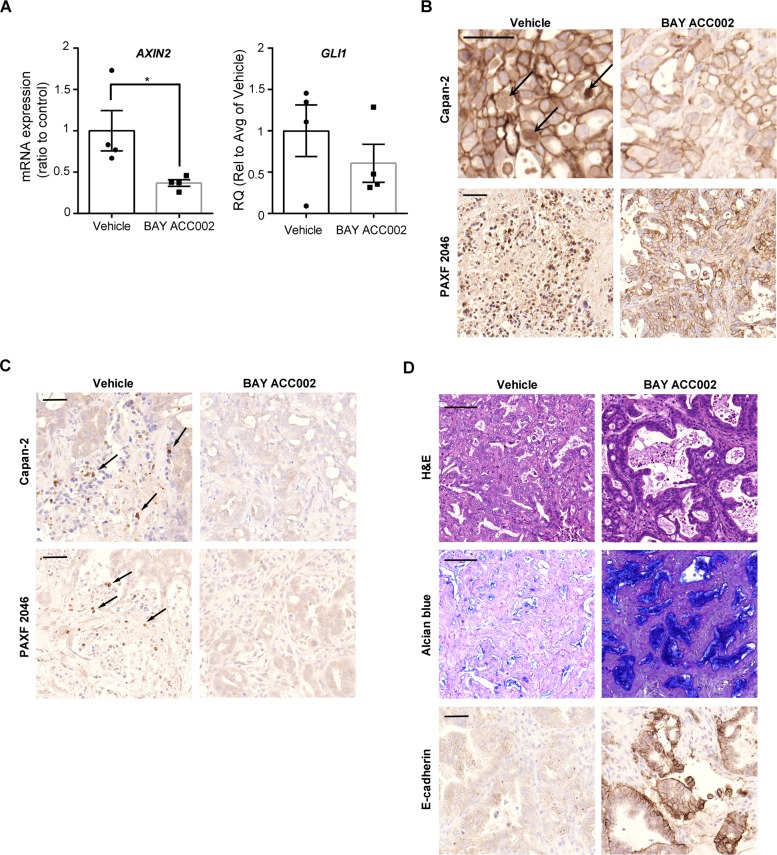
BAY ACC002 Decreases WNT and HH Signaling in Pancreatic Cancer Models *In Vivo* **A**. *AXIN2* and *GLI1* expression in Capan-2 pancreatic cancer tumors, treated with vehicle or 30 mg/kg/day BAY ACC002 for 7 days. RNA was extracted from the tumors and expression of *AXIN2* and *GLI1* was determined by qRT-PCR. Each bar represents mean±SEM, with individual animals represented by dots (*n* = 4, *, *p* < 0.05). **B**. IHC analysis of β-catenin expression in representative pancreatic cancer sections from mice, carrying Capan-2 or PAXF 2046 tumors, treated with vehicle or BAY ACC002 (35 mg/kg/day on days 1-5, and then 30 mg/kg/day on a 3 days ON/1 day OFF schedule until day 35 for the Capan-2 model; and 30 mg/kg/day for 29 days for the PAXF 2046 model). Arrows point to nuclear β-catenin expression. **C**. IHC analysis of GLI1 expression in representative pancreatic cancer sections from Capan-2 and PAXF 2046 tumors described in (B). Arrows point to GLI1 positive cells. **D**. H&E, Alcian blue, and E-cadherin IHC staining of representative sections from the PAXF 2046 tumors, described in (B). Scale bar for (B), (C), and the third set of (D) is 50 μm, and for the first two sets of (D), 100 μm.

We next tested the effect of ACC inhibition on WNT and HH signaling *in vivo*. Treatment with BAY ACC002 (30 mg/kg/day for 7 days p.o.) in a Capan-2 pancreatic cancer xenograft mouse model suppressed mRNA expression of the WNT/β-catenin target gene *AXIN2* by ∼60% and of the HH target gene *GLI1* by 40% (Figure 3A). Furthermore, immunohistochemical (IHC) analysis revealed that long-term treatment with BAY ACC002 in the same tumor model resulted in inhibition of transcriptional β-catenin activity, associated with decreased nuclear localization of β-catenin (Figure 3B, top). Even more striking effects were observed with BAY ACC002 in the PAXF 2046 pancreatic patient-derived xenograft (PDX) model, with moderate differentiation and high stromal content (57%) (Figure 3B, bottom). In both the Capan-2 and PAXF 2046 xenograft tumors IHC analysis also revealed that the expression of GLI1 was decreased following BAY ACC002 treatment, thus indicating simultaneous downregulation of HH pathway activity (Figure 3C). Furthermore, the effect of the ACC inhibitor on WNT and HH signaling was comparable to the effect of LGK974 on the WNT pathway and of GDC-0449 on the HH pathway, respectively (Supplementary Figure S3). Together these data demonstrate that inhibition of ACC can result in a dual inhibition of WNT and HH signaling in pancreatic tumors *in vivo*.

### Inhibition of ACC in vivo reverts cells to histologically epithelial phenotype

As BAY ACC002 could effectively suppress WNT signaling *in vivo*, we sought to investigate whether it could affect cell proliferation and differentiation in tumors - a phenotype, associated with WNT pathway inhibition (Supplementary Figure S3D) [17]. First, we demonstrated that short-term treatment with BAY ACC002 affected cancer cell proliferation *in vivo*, as revealed by a decrease in Ki-67 positive cells (Supplementary Figure S4A, S4B). Next, we investigated whether ACC inhibition also induced tumor cell differentiation. Hematoxylin and eosin (H&E) staining of PAXF 2046 xenograft tumors treated with the ACC inhibitor revealed an increase of ductal epithelium morphology compared to the moderately differentiated tumors observed in the vehicle group (Figure 3D). Furthermore, BAY ACC002 increased mucin production, as evidenced by an increase in Alcian blue staining, and increased expression of the epithelial phenotype marker E-cadherin (Figure 3D), thus suggesting that the tumor cells have indeed reverted to histologically epithelial phenotype. None of these changes in the tumors were observed in mice treated with the HH inhibitor GDC-0449 (Supplementary Figure S3D). Thus, ACC inhibition could induce reversion of pancreatic tumor cells to an epithelial phenotype, in line with its inhibition of WNT signaling.

### ACC inhibition suppresses tumor growth in several different pancreatic cancer xenograft models

**Figure 4:**
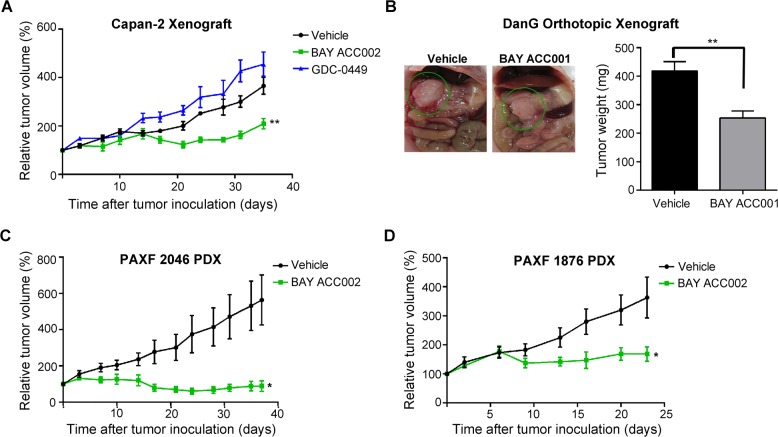
Sensitivity of Pancreatic Tumors to ACC Inhibition *In Vivo* **A**. Growth curves of Capan-2 tumors, treated with vehicle, BAY ACC002 (35 mg/kg/day on days 1-5, and then 30 mg/kg/day on a 3 days ON/1 day OFF schedule until the end of the study), or GDC-0449 (75 mg/kg, twice daily) for 35 days. Tumor volume was measured every three days. Graphs represent relative tumor volumes (mean±SEM) over time, with each point showing the (*n* = 5-6, **, *p* < 0.01 between vehicle and BAY ACC002 at the end point). **B**. Representative photographic images and tumor weights of DanG orthothopic pancreatic tumors, treated with vehicle or BAY ACC001 (30 mg/kg) twice daily for 12 days. At the time of sacrifice, pictures were taken and tumor weight was measured. Bars represent mean±SEM (n = 3-4, **, *p* < 0.01). **C**. Growth curves of PAXF 2046 PDX tumors treated with vehicle or BAY ACC002 (35 mg/kg/day) for 35 days. Tumor volume was measured every three days. Graphs represent relative tumor volumes (mean±SEM) over time (*n* = 5-6, *, *p* < 0.05). **D**. Growth curves of PAXF 1876 PDX tumors, treated with vehicle or BAY ACC002 (35 mg/kg/day on days 1-9 and 12-23). Tumor volume was measured every 3 days. Graphs represent relative tumor volumes (mean±SEM) over time (*n* = 7-8 *, *p* < 0.05 between vehicle and BAY ACC002 at the end point).

To assess the anti-tumor activity of ACC inhibition on pancreatic cancer, we tested BAY ACC002 in several different pancreatic cancer xenograft mouse models at a well-tolerated dose (body weight loss < 10%). In a Capan-2 pancreatic cancer mouse model, treatment with BAY ACC002 resulted in a significant tumor growth inhibition (TGI 42.6%, p < 0.01) at the end of the study. Meanwhile, despite suppressing HH/GLI1 signaling (Supplementary Figure S3C), the HH pathway inhibitor GDC-0449 did not show anti-tumor activity (Figure 4A). Given the importance of the tumor microenvironment in pancreatic cancer, we also tested the efficacy of ACC inhibition in an orthotopic tumor model, in which DanG pancreatic cancer cells were injected directly into the mouse pancreas parenchyma. Treatment of these animals with another ACC inhibitor (BAY ACC001) from the same compound class (Supplementary Figure S1A; Liu *et al*., in preparation) led to a 39% reduction in tumor weight (p < 0.01, Figure 4B). Additionally, in order to test the ACC inhibitors in tumor models with more heterogeneity and better mimicking patient response, we treated ten different subcutaneous PDX pancreatic cancer models (Supplementary Figure S4). 6/10 animals showed strong response with TGI>50% as single agent. PAXF 1876 and PAXF 2046 were amongst the best responders (Figure 4C, 4D), with BAY ACC002 blocking tumor growth by 53.6% (p < 0.05) in PAXF 1876. In the PAXF 2046 model, the effect was even more pronounced, resulting in tumor regression in 3/5 animals and an overall TGI of 83.4% (p < 0.05, Figure 4C). In the same model treatment with GDC-0449 showed no anti-tumor efficacy and was associated with increased *AXIN2* expression at the end of the study (Supplementary Figures S4D, S4E). Taken together, these findings demonstrate that ACC inhibition can effectively block pancreatic tumor growth *in vivo* as a single agent.

## DISCUSSION

Upregulation of *de novo* lipid biosynthesis is one of the hallmarks of cancer and cancer cells depend on it irrespective of exogenous lipid levels [11, 18, 19]. This upregulation is driven by overexpression of different proteins that are part of the pathway, and ACC, a rate-limiting enzyme of *de novo* fatty acid synthesis, is amongst them [11, 19, 20]. Compared to other fatty acids in tumor cells palmitate levels are most sensitive to ACC inhibition (Liu *et al*., in preparation). In turn, ligand palmitoylation has been described to be key regulator of the signaling activity of pathways such as WNT and HH [6, 7, 8]. In this paper we demonstrate for the first time that inhibition of ACC could suppress protein palmitoylation and could subsequently block oncogenic protein signaling. Using BAY ACC002, a highly selective ACC inhibitor, we showed that ACC inhibition decreased the level of palmitoylation of WNT3A, and prevented its secretion and signaling (Figure 1). Several lines of evidence supported the specific effect of ACC inhibition on modulation of WNT signaling. First, BAY ACC002 and the structurally-different, allosteric ACC inhibitor Soraphen A had similar effect on WNT3A secretion (Figure 1C). Second, the effect of BAY ACC002 on WNT secretion was specific, with no effect on secretion of non-palmitoylated proteins (e.g. CYR61, Figure 1D) and no general effect on the growth of WNT-producing cells. Finally, in WNT-responsive cells the ACC inhibitor did not alter the exogenous WNT3A-induced activation of WNT signaling (Figures 1F, 2C). Together these data suggest that the molecular mechanism by which ACC inhibition blocks WNT signaling is by suppressing palmitoylation and secretion of WNT ligands.

Since HH ligand activity is also regulated by palmitoylation [7, 8], we speculated that ACC inhibition could also affect HH ligand palmitoylation and activity. To study the effects of ACC inhibition on both HH and WNT signaling, we focused on PDAC, which is among the most aggressive and difficult to treat cancers, and in which HH and WNT signaling are key oncogenic drivers, involved in the complicated signaling events between tumor and stromal cells [16, 17]. Indeed, in the Capan-2 pancreatic tumor cells, where autocrine WNT and HH signaling is active, BAY ACC002, as well as Soraphen A, suppressed both WNT and HH signaling *in vitro* (Figure 2A, 2D). *In vivo*, ACC inhibition also resulted in inhibition of both WNT and HH signaling, as evidenced by changes in β-catenin localization and GLI1 expression (Figure 3A-3C). Furthermore, BAY ACC002′s effect on the WNT pathway was partially rescued by addition of exogenous WNT3A to Capan-2 cells (Figure 2B), thus directly linking ACC inhibition to WNT signaling. However, full rescue in this case was not achieved possibly due to the involvement of other WNTs, such as WNT7B, in pathway activation [21]. Altogether, the data support the hypothesis that down-regulation of palmitate by ACC inhibition can simultaneously block WNT and HH palmitoylation, and consequently can successfully suppress both pathways in pancreatic cancer.

Functionally, ACC inhibition was accompanied by a decrease in proliferation in pancreatic cancer cells *in vitro* and *in vivo* (Figure 2F-2I, Supplementary Figures S4A, S4B). The anti-proliferative effect of BAY ACC002 was present in cell systems which have been validated by us and others to be dependent on autocrine WNT (Capan-2, Supplementary Figure S2E) and/or HH [17] signaling for their proliferation. Thus, for example, in L-Wnt3A cells, where WNTs are not the drivers of proliferation, ACC inhibition led to suppression of WNT3A lipidation, secretion, and signaling (Figure 1), but did not show any effect on cell proliferation (Supplementary Figure S1D). In addition, the anti-proliferative effect of the ACC inhibitor appeared only after a significant inhibition of HH and WNT signaling was achieved (Figure 2A, 2D, 2G). Together, these data suggest that modulation of oncogenic signaling, such as WNT and HH, and not of structural membrane lipid components, is linked to the anti-proliferative effects observed with ACC inhibition in pancreatic cancer cells. At this point, however, we cannot conclude if only WNT and HH, and no additional signaling molecules, are associated with the ACC inhibition-mediated anti-proliferative effect.

In this study, we demonstrated that treatment with ACC inhibitors resulted in significant tumor growth inhibition in a wide variety of cell line-based and patent-derived xenograft models *in vivo* (Figure 4, Supplementary Figure S4C). Notably, ACC inhibition revealed clear anti-tumor activity in Gemcitabine-resistant models such as PAXF 2046, where BAY ACC002 caused significant tumor regression, while Gemcitabine showed only moderate tumor growth inhibition (Supplementary Figure S4C). Furthermore, BAY ACC002 was active in both mutant *KRAS* (Capan-2, DanG) and wild-type *KRAS* (BxPC-3) pancreatic cancer cells. Additionally, BAY ACC002 showed efficacy in tumors where the HH inhibitor GDC-0449 was ineffective (Figure 4A, Supplementary Figure S4D). Accumulating evidence suggests that the lack of efficacy of the latter could be in part due to crosstalk between HH and WNT signaling. Here, we demonstrate that *SFRP1*, a negative regulator of WNT signaling and a point of crosstalk between the two pathways [22], was upregulated in pancreatic tumors (Supplementary Figure S2B). Furthermore, treatment with GDC-0449 decreased *SFRP1* expression, while maintaining elevated levels of WNT response genes in Capan-2 cells *in vitro* (Supplementary Figure S3E), and increasing *AXIN2* expression in the PAXF 2046 model *in vivo* (Supplementary Figure S4E). These effects were not observed with ACC inhibitors, where both HH and WNT signaling were inhibited (Figures 2A, 2D, 3A). Taken together, these observations highlight the potential broad utility of ACC inhibitors for pancreatic cancer treatment.

In summary, this study suggests that *de novo* lipid biosynthesis in tumor cells may have a primary impact on oncogenic signaling rather than on membrane structural lipids. We demonstrate for the first time that ACC inhibition, in particular, could be a viable approach to modulate the HH and WNT oncogenic signaling pathways via regulation of palmitoylation and activation of their ligands. In addition, we show that ACC inhibitors could offer a novel approach for the treatment of PDAC, where HH and WNT oncogenic pathways are frequently dysregulated. Furthermore, since palmitoylation modulates the membrane localization and activation of many oncogenic signaling proteins, such as RAS proteins [23], as well as estrogen, androgen and progesterone receptors [24], ACC inhibition might offer a promising new approach for the treatment of tumors with RAS mutations and/or altered activation of hormone receptors. Overall, our findings highlight a potential new strategy of using ACC inhibitors for cancer treatment and invite for further investigation on targeting protein lipidation for the treatment of cancer and other diseases.

## MATERIALS AND METHODS

### Compounds

BAY ACC001 and BAY ACC002 were identified and synthesized at Bayer AG (Leverkusen, Germany) (Liu et al., in preparation). LGK974 (S7143) and GDC-0449 (S1082) were purchased from Selleckchem (Houston, TX, US).

### Cell lines, reagents and antibodies

All cancer cell lines, as well as the L and L-Wnt3A cells, were obtained from the American Type Culture Collection (Manassas, VA, US), maintained according to their guidelines and authenticated by fingerprint techniques at German Collection of Microorganisms and Cell Cultures (DSMZ).

SHH (C9C5) Rabbit monoclonal antibody (2207), non-phospho β-catenin (Ser33/37/Thr41) (D13A1) rabbit monoclonal antibody (8814), and AXIN2 (76G6) rabbit monoclonal antibody (2151) were purchased from Cell Signaling (Danvers, MA, US). Anti-WNT3A rabbit polyclonal antibody (09-162) was purchased from Millipore. GLI1 (H-300) rabbit polyclonal antibody (sc-20687) and Cyr61 (H-78) rabbit polyclonal antibody (sc-13100) were purchased from Santa Cruz Biotechnology Inc. (Santa Cruz, CA, US). E-cadherin mouse monoclonal antibody (MA5-12547) was acquired from Thermo Fisher Scientific (Waltham, MA, US). Dako Monoclonal Mouse anti-Human Ki-67 Clone MIB-1 was obtained from Dako (Hamburg, Germany). Protein G Sepharose was purchased from GE Healthcare (Little Chalfont, UK).

Recombinant human WNT3A protein (5036-WN) was purchased from R&D Systems (Minneapolis, MN, US).

Pancreatic tissue micro array (TMA) was purchased from Oncotest GmbH.

### Plasmids and transfection

PORCN human cDNA plasmid (SC316376) was purchased from OriGene (Rockville, MD, US). For cell transfections with plasmids, Lipofectamine LTX Plus transfection reagent (Thermo Fisher Schientific) was used and the manufacturer's instructions were followed.

### Luciferase assay

Conditioned media from L-Wnt3A cells, treated with vehicle or BAY ACC002 for 72 h, was collected and added to HEK293 cells, stably expressing TOPFlash reporter (established at Bayer AG). 48 h later WNT pathway activity was measured using ONE-Glo luciferase assay system (Promega, Madison, WI, US).

### Proliferation assays

DanG, Capan-2, BxPC-3 and Panc-1 cells were seeded and 24 h later, the media was changed to media, containing 5% charcoal-stripped FCS and varying concentrations of BAY ACC002: 0.04, 0.12, 0.37, 1.1, 3.3, and 10 μM for DanG cells, and 1 and 10 μM for Capan-2, BxPC-3 and Panc-1 cells. Cell proliferation was measured using the xCELLigence system (Roche), which provides real-time data on cell viability and proliferation, using electrical impedance as readout.

### qRT-PCR

Total RNA was isolated from cells or tissue with the InviTrap Spin Cell or Spin Tissue RNA Mini Kit (Stratec, Berlin, Germany) according to the manufacturer's instructions. cDNA was synthesized using the SuperScript III First-Strand Synthesis SuperMix for qRT-PCR (Invitrogen) and following the manufacturer's instructions. qRT-PCR was used to determine gene expression levels using TaqMan Universal PCR Mastermix (Applied Biosystems, Foster City, CA, US); individual TaqMan probes for the different genes of interest, and custom-made spotted 384-well plates; and the HT9700 Real-Time Fast Cycler (Applied Biosystems). GAPDH was used as an endogenous reference gene, and the results were analyzed with the DataAssist Software (Applied Biosystems).

### Triton X-114 phase separation assay

L-Wnt3A cells were seeded and transfected with PORCN plasmid on the next day. After 24 h, the media was replaced with media containing 2% charcoal-stripped fetal calf serum (FCS) and DMSO, BAY ACC002 or LGK974. After 24 h of treatment, the cells were washed with ice-cold phosphate-buffered saline (PBS) and were incubated for 15 min on ice in Lysis Buffer (10 mM Tris-HCl pH 7.5, 150 mM NaCl, 1% TritonX-114, cOmplete^TM^ Mini Protease Inhibitor Cocktail tablet (Roche, Switzerland), PhosStop tablet (Roche). The cells were scraped, and the lysate was cleared for 10 min at 13000 g at 4°C. The supernatant was divided in two fractions. One was kept as the input (its volume was brought up to 1 mL using Lysis Buffer) and the other was diluted with equal parts of Lysis Buffer and Modified Lysis Buffer, containing 3.5% TritonX-114. After 15 min incubation at 4°C with rotation, the samples were placed for 5 min in a 37°C water bath, and subsequently centrifuged at 2000 g for 5 min at room temperature. The aqueous layer (top) and the lipid layer (bottom) were collected separately and their volumes were brought up to 1 mL using Lysis Buffer. Next, WNT3A was immunoprecipitated from each sample. WNT3A was immunoprecipitated by incubating samples with a rabbit polyclonal WNT3A antibody (Millipore) and Protein G Sepharose beads (GE Healthcare) overnight at 4°C with rotation. The samples were washed five times in PBS, containing 0.02% Tween 20, and finally resuspended in 30 μL DTT-supplemented NuPAGE LDS Sample Buffer (Invitrogen, Carlsbad, CA, US), and heated up at 70°C for 10 min. WNT3A protein levels were subsequently detected by Western blot.

### Secretion assay

Cells were seeded and 24 h later, switched to media, containing 2% charcoal-stripped FCS and either DMSO, BAY ACC002 or LGK974. After 48 h, conditioned media was cleared by centrifugation at 500 *g* for 5 min and concentrated to final volume of 100 μL using 10,000 molecular weight cutoff centrifugal devices (Sartorius Stedim Biotech, Concord, CA, US). The amount of WNT3A in the media was analyzed by Western blot.

### Immunoblotting

Cells were washed with ice-cold PBS and lysed in M-PER Mammalian Protein Extraction Reagent (Thermo Fisher), supplemented with cOmplete™ Mini Protease Inhibitor Cocktail tablet (Roche) and a PhosStop tablet (Roche). The cell lysate was cleared by ultracentrifugation and samples were diluted in NuPAGE LDS Sample Buffer. Samples were run on NuPAGE Novex 4-12% Bis-Tris Gels (Invitrogen) and then transferred onto polyvinylidene fluoride (PVDF) membrane. The membranes were then incubated with the appropriate antibodies overnight at 4°C, washed, and then incubated with secondary antibody for 1 h at room temperature. Membranes were next incubated for 5min in Pierce ECL Plus Western Blotting Substrate (Thermo Fisher), and exposed to Hyperfilm ECL autoradiography film (Amersham, Little Chalfont, UK) to detect proteins. Protein levels were quantified using ImageJ.

### *In vivo* studies

The *in vivo* antitumor efficacy and mechanism of action of ACC inhibitors were assessed in 5-8-week-old female SCID mice (Envigo, Cambridgeshire, UK), using cell-line derived (Capan-2 and DanG) and patient-derived xenografts of human pancreatic tumors. Studies on Capan-2 and PDX subcutaneous tumor models were conducted at Oncotest GmbH (Freiburg, Germany) and the DanG orthotopic tumor model study at Bayer AG.

Tumor cell fragments were injected subcutaneously into the right flank of each mouse in the Capan-2, PAXF 1876, and PAXF 2046 models. In the orthotopic DanG study, the cells were inoculated into the pancreas parenchyma [25, 26].

BAY ACC002 was dissolved in PEG400 / ethanol / Solutol^®^ HS 15 (70/5/25), BAY ACC001 was dissolved in N-Methyl-2-pyrrolidin (NMP) / PEG300 (1:9), GDC-0449 was dissolved in 0.5% methylcellulose, 0.2% polysorbate 80, and LGK-974 was dissolved in 0.5% methylcellulose, 0.5% Tween80. PEG400 / ethanol / Solutol^®^ HS 15 (70/5/25) was used as the vehicle in the Capan-2, PAXF 1876 and PAXF 2046 studies, while NMP / PEG300 (1:9) was used as the vehicle for the DanG study. The mice were randomized based on the tumor area and the treatment was initiated when all animals in an experiment had established tumors.

In the Capan-2 xenograft mouse model, vehicle or BAY ACC002 was administered orally either at 30 mg/kg/day for 7 days, or at 35 mg/kg/day on days 1-5, and then at 30 mg/kg/day on a 3 days ON/1 day OFF schedule until day 35. In the latter study, GDC-0449 was administered twice daily at 75 mg/kg (p.o.).

In the DanG xenograft model, mice were administered vehicle or BAY ACC001 (30 mg/kg, p.o.) twice daily for 12 days. All mice bearing PDX models, except PAXF 1876 and PAXF 2046, were treated with BAY ACC002 (35 mg/kg/day, p.o.) daily for 23-35 days. Mice bearing PAXF 1876 xenograft tumors were treated with BAY ACC002 (35 mg/kg/day, p.o.) daily on days 0-9 and 12-23. In the PAXF 2046 xenograft model, the animals were treated with BAY ACC002 at 35 mg/kg/day, p.o. for 35 days or with 30 mg/kg/day, p.o. for 29 days. In the latter study, GDC-0449 was administered twice daily at 75 mg/kg (p.o.) and LGK974 was administered twice daily at 5 mg/kg (p.o.) for 12 days, and then twice at 4 mg/kg/day until day 29.

Tumor dimensions and body weights were recorded twice weekly starting on the first day of treatment. Tumor volumes were calculated using the equation: 0.5 x length x width^2^. Tumor growth inhibition (TGI) was calculated by the equation (1-T/C) x100, where *T* and *C* represent the mean size of tumors in the treated (T) and control (C) groups, respectively. At the endpoint of the studies, the tumors were collected 6 h or 24 h after the final dosing and were further processed for *ex vivo* analysis by qRT-PCR and immunohistochemistry. For the orthothopic study, pictures of tumors in the pancreas were taken and tumor weight was measured at the time of sacrifice.

All animal experiments were performed under German Animal Welfare Law and approved by local authorities. For studies with PDX models, written informed consent from each patient and the approval from local ethical committees were obtained.

### Immunohistochemistry

Formalin-fixed, paraffin-embedded tumor samples were cut into 3μm sections, subsequently stained with hematoxilin and eosin (H&E), as well as Alcian blue. The sectioning and staining were performed at the Institut fuer Tierpathologie (Berlin, Germany).

For antibody staining, the sections were rehydrated and antigen retrieval was performed by incubating the slides in Dako Target Retrieval solution, pH 6 (for GLI1 and Ki-67antibodies) or Dako Target Retrieval solution, pH 9 (for β-catenin and E-cadherin antibodies) for 40 min in a steamer. After cooling the slides for 40 min, they were washed in a buffer consisting of Tris-buffered saline and Tween20 (TBST) and incubated with Dako Peroxidase Blocking Reagent for 15 min. The slides were then washed with TBST and incubated with Dako Protein Block for 1 h. This was followed by incubation with the respective primary antibodies for 1 h at room temperature, at concentrations recommended by the manufacturer. After this, the slides were processed further to detect the primary antibodies, using the Dako EnVision+ System-HRP (DAB) system, designed for rabbit or mouse primary antibodies, and following the manufacturer's instructions. Finally, the sections were dehydrated and cover-slipped for microscopic observation. Stained slides were digitally scanned using Mirax MIDI BF/FL Digital Slide Scanner (Karl Zeiss AG, Oberkochen, Germany), and analyzed using Panoramic Viewer (3DHistech, Budapest, Hungary).

### Statistical analysis

All data are presented as mean±SEM and the n for each experiment is indicated in the figure legend. The analysis was performed using GraphPad Prism software and applying a Student's *t* test (unpaired, two-tailed), and results were considered significant at *p*-value < 0.05.

## SUPPLEMENTARY MATERIALS FIGURES


