# Supplementary material for: Acetyl-CoA carboxylase inhibitors attenuate WNT and Hedgehog signaling and suppress pancreatic tumor growth
Source: Oncotarget. 2016 Oct 13;8(30):48660–70. doi: 10.18632/oncotarget.12650 (PMC5564715; doi:10.18632/oncotarget.12650)
Supplement: Supplementary file 1 [file oncotarget-08-48660-s001.pdf]

Acetyl-CoA carboxylase inhibitors attenuate WNT and Hedgehog signaling and suppress pancreatic tumor growth

Supplementary Material

Supplementary Figure S1

A

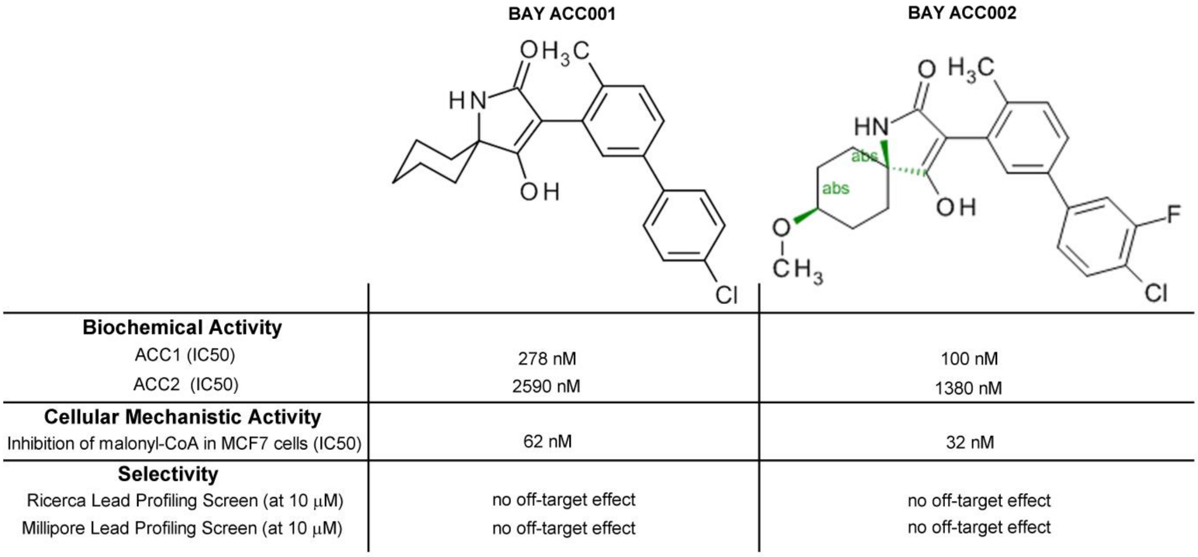

	BAY ACC001	BAY ACC002
**Biochemical Activity**		
ACC1 (IC50)	278 nM	100 nM
ACC2 (IC50)	2590 nM	1380 nM
**Cellular Mechanistic Activity**		
Inhibition of malonyl-CoA in MCF7 cells (IC50)	62 nM	32 nM
**Selectivity**		
Ricerca Lead Profiling Screen (at 10 μM)	no off-target effect	no off-target effect
Millipore Lead Profiling Screen (at 10 μM)	no off-target effect	no off-target effect

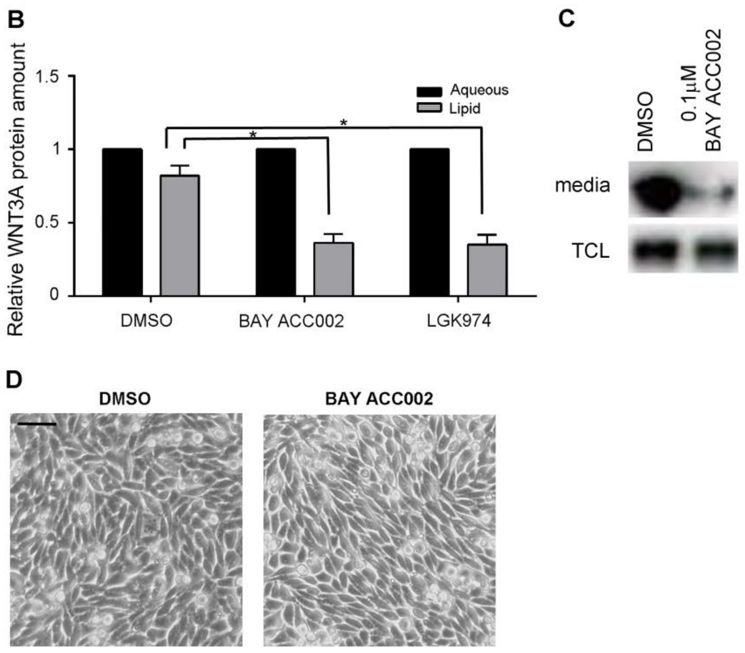

Supplementary Figure S1: Structure and Activity of ACC Inhibitors

(A) Structure, activity and selectivity of BAY ACC001 and BAY ACC002. Biochemical activity of the inhibitors against human isoforms ACC1 and ACC2 is reported as IC50 values obtained in an assay monitoring ACC-mediated generation of ADP using recombinant proteins. The cellular mechanistic activity is reported as IC50 values determined by measuring inhibition of malonyl-CoA levels in MCF7 cells. The specificity of the compounds was investigated by measuring their activity at 10 µM against 68 potential off-targets (receptors, channels, transporters, plus the fatty acid-related enzymes acyl-CoA-cholesterol acyltransferase, aldose reductase, carnitine palmitoyltransferase-1 (CPT-1), fatty acid amide hydrolase (FAAH)) (Ricerca, Lead Profiling Screen) in a biochemical radioligand binding assay, as well as by measuring their activity at 10 µM versus a panel of 222 kinases (Millipore, Lead Profiling Screen). In both profiling screens >50% inhibition was the cut-off for significant off-target activity.

(B) Relative WNT3A levels in three independent experiments as described in Figure 1A. The amount of WNT3A in the lipid phase was normalized to the amount of WNT3A in the aqueous phase for each sample. Each bar represents mean±SEM (n=3, *, $p<0.05$, Student's *t* test).

(C) Amount of WNT3A secreted from cells treated with DMSO or 0.1 µM BAY ACC002 for 48 h. Amount of WNT3A in the media and in the TCL was quantified by Western blot.

(D) Effect of BAY ACC002 on L-Wnt3A cell growth. Phase-contrast images of the L-Wnt3A cells treated with DMSO and BAY ACC002 from the experiment described in 1C. Scale bar is 100 µm.

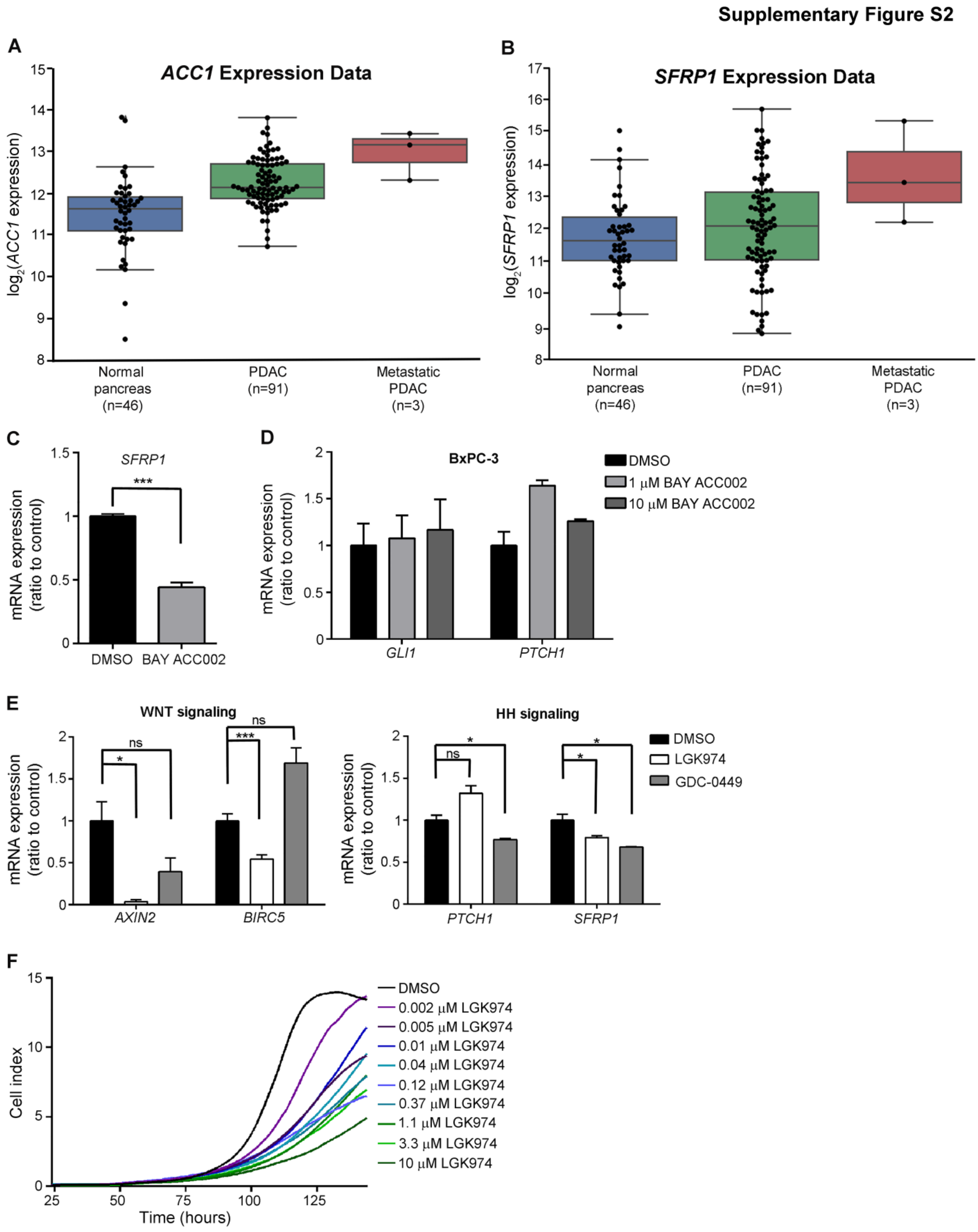

Supplementary Figure S2: ACC1 and SFRP1 Expression in Pancreatic Cancer, and Dependency of Pancreatic Tumors on WNT Ligand Production

(A) *ACC1* expression in normal (n=46), PDAC (n=91), and metastatic PDAC (n=3) pancreatic patient samples were analyzed by an Affymetrix GeneChip assay (probe set 212186_at). The center of each box represents the median, with the lower and upper limit of the box representing the 25th and 75th quantiles. To determine the position of the whiskers 1.5 x box length is added at each side of the box as initial position of whiskers, and then dragged towards the box until they hit a real value. All values outside the whiskers are outliers. Each dot represents an individual sample.

(B) *SFRP1* expression in normal (n=46), PDAC (n=91), and metastatic PDAC (n=3) patient samples analyzed by an Affymetrix GeneChip assay (probe set 202037_s_at). The center of each box represents the median, with the lower and upper limit of the box representing the 25th and 75th quantiles. To determine the position of the whiskers 1.5 x box length is added at each side of the box as initial position of whiskers, and then dragged towards the box until they hit a real value. All values outside the whiskers are outliers. Each dot represents an individual sample.

(C) *SFRP1* expression levels in Capan-2 cells, treated with DMSO or 10 μM BAY ACC002 for 72 h.

(D) Expression levels of the β-catenin target genes *AXIN2* and *BIRC5* (left) and the HH target genes *PTCH1* and *SFRP1* (right) in Capan-2 cells, treated with DMSO, 5 μM LGK974 (PORCN inhibitor), or 10 μM GDC-0449 (SMO inhibitor) for 56 h (left) or 48 h (right).

(E) Effect of LGK974 on Capan-2 pancreatic cancer cell proliferation. Cell growth was measured over time using the xCELLigence system. Experiment was performed in triplicate.

In (C) and (D) each bar represents mean±SEM (n=3). ns, non-significant, *, p<0.05, ***, p<0.001 Student's *t* test compared to DMSO.

Supplementary Figure S3

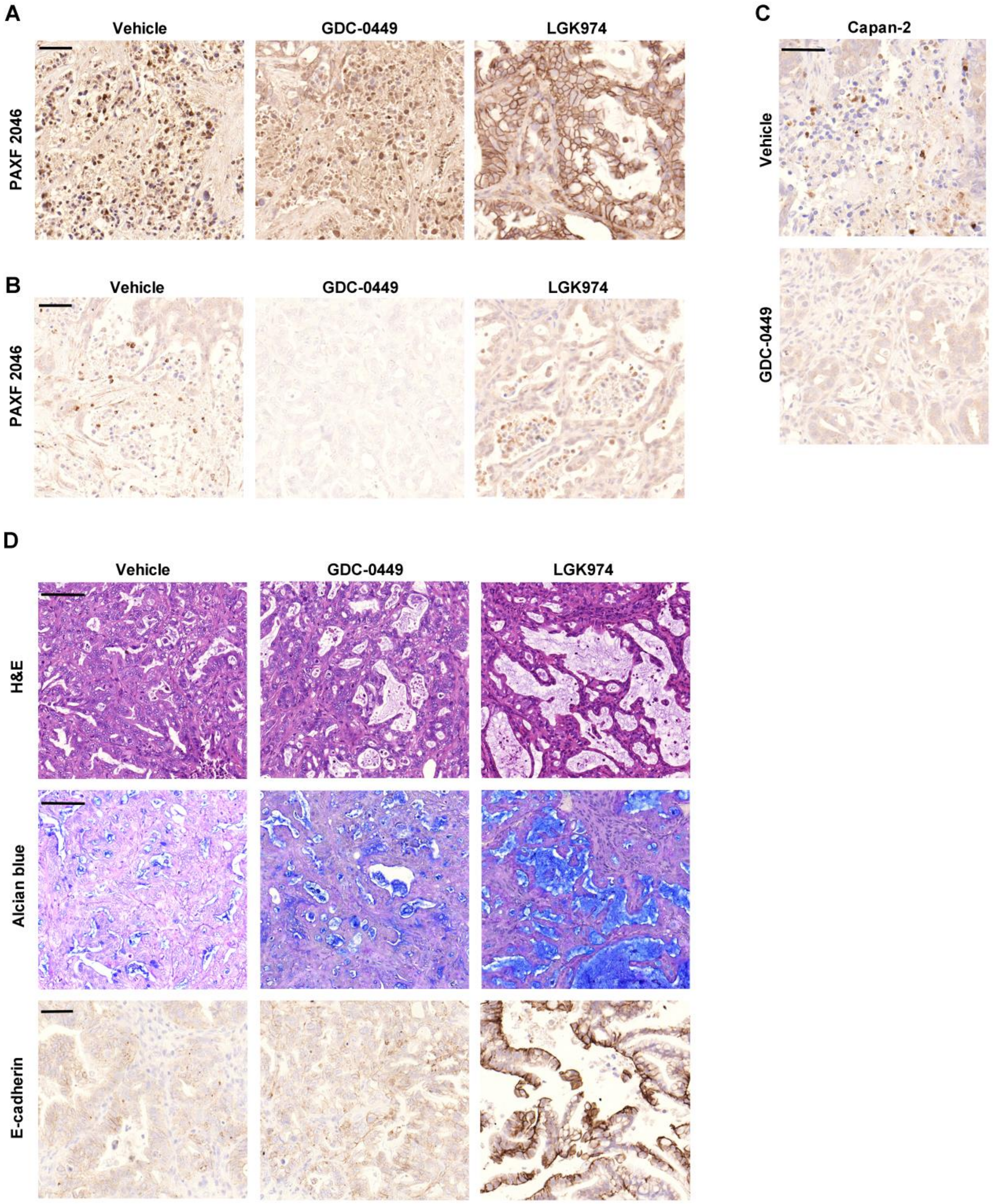

Supplementary Figure S3: Effect of GDC-0449 and LGK974 on WNT and HH Signaling in Pancreatic Cancer Cells *In Vitro* and *In Vivo*

(A) IHC analysis of β-catenin expression in representative tissue sections from PAXF 2046 pancreatic cancer PDX tumors, treated with vehicle, GDC-0449 (75mg/kg, twice daily for 29 days) or with LGK974 (twice daily at 5 mg/kg for 12 days, and then twice daily at 4 mg/kg until day 29). Scale bar is 50 µm.

(B) IHC analysis of GLI1 expression in tissue sections described in (A). Scale bar is 50 µm.

(C) IHC analysis of GLI1 expression in tissue sections from Capan-2 pancreatic cancer xenograft tumors, treated with vehicle or GDC-0449 (75mg/kg) twice daily for 35 days. Scale bar is 50 µm.

(D) H&E stained, Alcian blue stained and IHC analysis of E-cadherin expression in tissue sections described in (A). Scale bars are 100 µm in the top panels and 50 µm in the bottom panel.

Supplementary Figure S4

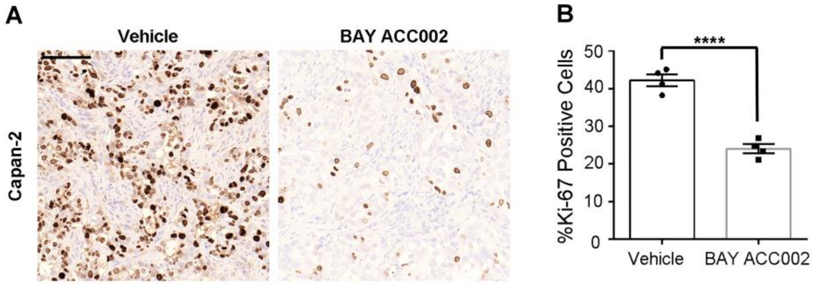

C

Tumor model	Efficacy (T/C (%)) BAY ACC002	Gemcitabine
PAXF 1982	8(4/5 PR)	36*
PAXF 2046	18(3/5 PR)	48
PAXF 1965	34	35
PAXF 1876	46	66
PAXF 2035	43	ND
PAXF 1986	44	58*
PAXF 1900	56	45*
PAXF 1998	57	5*
PAXF 1861	63	48
PAXF 546	72	57*

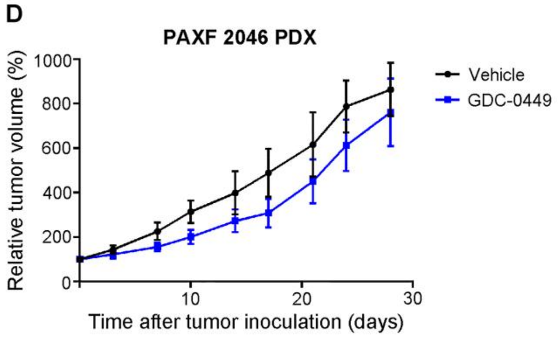

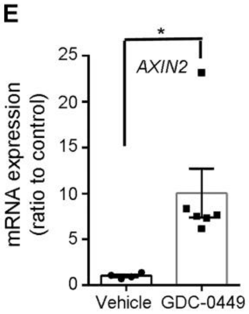

Supplementary Figure S4: Effect of ACC Inhibition on Pancreatic Cancer Cell Proliferation and Effect of GDC-0449 on Pancreatic Tumor Growth

(A) IHC analysis of Ki-67 in tissue sections from Capan-2 pancreatic cancer xenografts, treated with vehicle or BAY ACC002 (30mg/kg/day) for 7 days.

(B) The percentage of Ki-67 positive cells in the experiment described in (D). The percentage of Ki-67 positive and negative cells was determined for each animal by counting the Ki-67 positive and negative cells in five representative 30X fields, and the average for all animals in each treatment group was plotted.

(C) Summary of anti-tumor efficacy of BAY ACC002 and gemcitabine in pancreatic PDX tumor models. Animals were treated with vehicle, BAY ACC002 (35 mg/kg daily; except for PAXF 1876, where treatment was on days 0-9 and 12-23) or Gemcitabine (100 mg/kg every 4 days). Anti-tumor efficacy was calculated at the end of the study using treatment (T) versus control (C). * Historical data in the same PDX model.

(D) Growth curves of PAXF 2046 PDX tumors treated with vehicle or GDC-0449 (75 mg/kg) twice daily for 29 days. Tumor volume was measured every three days. Graph represents relative tumor volumes (mean±SEM) over time (n=6).

(E) *AXIN2* expression in Capan-2 pancreatic cancer tumors, treated with vehicle or GDC-0449 (75 mg/kg, twice daily) for 35 days. RNA was extracted from the tumors and expression of *AXIN2* was determined by qRT-PCR.

In (B) and (E), each bar represents mean±SEM, with individual animals represented by dots (n=4-6, *, $p<0.05$, ****, $p<0.0001$, Student's *t* test).
